# Elucidating genes and gene networks linked to individual susceptibility to milk fat depression in dairy goats

**DOI:** 10.3389/fvets.2022.1037764

**Published:** 2022-12-15

**Authors:** Aroa Suárez-Vega, Beatriz Gutiérrez-Gil, Pablo G. Toral, Pilar Frutos, Juan J. Loor, Juan-José Arranz, Gonzalo Hervás

**Affiliations:** ^1^Departamento de Producción Animal, Facultad de Veterinaria, Universidad de León, León, Spain; ^2^Instituto de Ganadería de Montaña (CSIC-Universidad de León), León, Spain; ^3^Department of Animal Sciences, Division of Nutritional Sciences, University of Illinois, Urbana, IL, United States

**Keywords:** goat, milk-fat depression, RNA-Seq, nutrigenomics, individual susceptibility, WGCNA

## Abstract

Dietary supplementation with marine lipids modulates ruminant milk composition toward a healthier fatty acid profile for consumers, but it also causes milk fat depression (MFD). Because the dairy goat industry is mainly oriented toward cheese manufacturing, MFD can elicit economic losses. There is large individual variation in animal susceptibility with goats more (RESPO+) or less (RESPO–) responsive to diet-induced MFD. Thus, we used RNA-Seq to examine gene expression profiles in mammary cells to elucidate mechanisms underlying MFD in goats and individual variation in the extent of diet-induced MFD. Differentially expression analyses (DEA) and weighted gene co-expression network analysis (WGCNA) of RNA-Seq data were used to study milk somatic cell transcriptome changes in goats consuming a diet supplemented with marine lipids. There were 45 differentially expressed genes (DEGs) between control (no-MFD, before diet-induced MFD) and MFD, and 18 between RESPO+ and RESPO–. Biological processes and pathways such as “RNA transcription” and “Chromatin modifying enzymes” were downregulated in MFD compared with controls. Regarding susceptibility to diet-induced MFD, we identified the “Triglyceride Biosynthesis” pathway upregulated in RESPO– goats. The WGCNA approach identified 9 significant functional modules related to milk fat production and one module to the fat yield decrease in diet-induced MFD. The onset of MFD in dairy goats is influenced by the downregulation of *SREBF1*, other transcription factors and chromatin-modifying enzymes. A list of DEGs between RESPO+ and RESPO– goats (e.g., *DBI* and *GPD1*), and a co-related gene network linked to the decrease in milk fat (*ABCD3, FABP3*, and *PLIN2*) was uncovered. Results suggest that alterations in fatty acid transport may play an important role in determining individual variation. These candidate genes should be further investigated.

## Introduction

Several feeding strategies have proven efficient in modulating ruminant milk composition toward a healthier fatty acid (FA) profile for consumers (1, 2). This includes, for instance, reductions in atherogenic saturated FA and increases in CLA, vaccenic acid or long chain n-3 polyunsaturated FA (3, 4). However, some of these strategies (namely, diet supplementation with marine lipids) can induce milk fat depression (MFD) in dairy ruminants, although goats are less prone than cows to this syndrome (2, 5). Because most caprine milk is used for cheese manufacturing, MFD is always related to economic losses in this species.

Interestingly, a large individual variation in the extent of diet-induced MFD has been observed in dairy ruminants (6, 7), including dairy goats from the same experimental flock when consuming a diet supplemented with 2% fish oil (8). When trying to improve milk fatty acid composition, understanding the genetic mechanisms behind this variability, which remain uncertain, would help explain individual variation in fish oil-induced MFD severity and select for less-susceptible animals.

The introduction of high-throughput technologies to study the genome provides ruminant scientists with a valuable tool to better understand animals' responses to nutrition, and it has boosted research in the field of nutrigenomics [i.e., the study of genome-wide influences of nutrition (9, 10)]. Technologies such as RNA-sequencing (RNA-Seq) along with bioinformatics allow for examining the expression of the entire set of transcribed genes in a sample, offering key information on the fundamental effects of dietary components on physiological outcomes (10). This approach has been applied for elucidating genes involved in mammary transcriptome responses to dietary lipid supplementation in cows and sheep (11–13). In dairy goats, less information on the effects of lipid supplementation on lipogenic gene expression is available (14); we are only aware of one *in vivo* study applying RNA-Seq to examine the transcriptomic changes associated with MFD in the mammary gland of goats fed different levels of degradable starch (15). However, in this species, MFD associated with dietary starch is a much less usual condition than marine lipid-induced MFD (1, 2, 8). Thus, the characterization of the transcriptomic changes underlying marine lipid-induced MFD would provide useful information to determine the genetic mechanisms influencing this complex trait. Moreover, the identification of genes and variants involved in the individual susceptibility to MFD in goats and their potential use as biomarkers could be useful to improve the classification of the animals and select those less susceptible to diet-induced MFD.

Differential gene expression analyses (DEA) have been successfully used to determine genes that are differentially expressed between experimental conditions (16, 17). However, the use of network approaches, such as the Weighted Gene Co-expression Network Analysis (WGCNA) method, has emerged as a useful alternative to elucidate the genetic basis of complex traits. These approaches do not aim to identify individual differentially expressed transcripts but clusters (modules) of highly co-expressed genes that are up/down regulated under certain biological, chemical or environmental conditions (18).

Based on the hypothesis that MFD in goats is partly driven by changes in the mammary gland gene expression profile, the aim of the present study was to use RNA-Seq data from the milk somatic cells (MSC) to identify genes and gene co-expression networks that differentiate distinct responses to diet induced MFD in goats.

## Materials and methods

### Animals, diets, and management

A detailed description of procedures, including animals, diets, and sampling, is provided by Della Badia et al. (8). All experimental procedures were performed in accordance with European Union and Spanish legislations (Council Directive 2010/63/EU and R. D. 53/2013). The animal use protocol was approved by the Research Ethics Committees of the *Instituto de Ganader*í*a de Montaña*, the Spanish National Research Council (CSIC), and the *Junta de Castilla y León* (Spain).

Briefly, 25 Murciano-Granadina goats (body weight: 30.5 ± 3.6 kg) at a similar stage of their first lactation (days in milk: 40.1 ± 5.9) were housed in individual tie stalls. During a 3 weeks adaptation period, goats were fed a TMR formulated with alfalfa hay (particle size > 4 cm) and concentrate (forage:concentrate ratio 50:50) without lipid supplementation [see Della Badia et al. (8) for details]. Subsequently, goats were supplemented with 20 g of fish oil (FO; Afampes 121 DHA, Afamsa)/kg of diet DM, which is known to cause MFD in dairy goats (8), for five additional weeks to induce a stable inhibition in milk fat synthesis. At the end of this second period, and on the basis of the magnitude of MFD, the five most-responsive goats (RESPO+; with a mean decrease in milk fat content of 26.0%) and the five least-responsive goats (RESPO–; with a mean decrease in milk fat content of 9.7%) were selected. Animals were milked once daily at ~08:30 h in a 10-stall milking parlor (DeLaval). They had *ad libitum* access to diets daily, and clean drinking water was available at all times.

### Sampling, RNA-extraction and RNA-sequencing

Milk samples for RNA extraction were collected at the end of the adaptation period (no-MFD, control samples) from all goats and at the end of the fish-oil (FO) supplementation period from goats selected as RESPO– and RESPO+. Sample collection and RNA extraction were performed according to Toral et al. (19), with little modifications. Milk somatic cells from healthy udders have been proven to represent the lactating mammary gland transcriptome in ruminants and a valid proxy to more invasive approaches, with correlations of 0.98 with mammary gland biopsies (20, 21). Moreover, this source of RNA has already been successfully applied in nutrigenomic studies in sheep to determine genes and pathways involved in MFD associated with FO and conjugated linoleic acid supplementation (11, 12). Briefly, milk samples were collected 1 h after milking and 10 min after injection of 5 IU of oxytocin/animal (Facilpart, Laboratorios SYVA) to maximize milk somatic cell concentration. To prevent RNA degradation, udders were cleaned with soap and water and disinfected with povidone-iodine, and the nipples were also flushed with RNAseZap (Ambion). A sterile gauze was used to cover the collection tube to avoid contamination.

For RNA extraction, the MSCs from 50 ml of fresh milk were pelleted by centrifugation at 650 × g for 10 min at 4°C in the presence of a final concentration of 0.5 mM of EDTA. Then, the pellet was washed three times with 10, 2, and 1.5 ml of PBS (pH 7.2 and 0.5 mM of EDTA), followed by centrifugation at 650 × g for 10 min at 4°C. The last pellet was kept in RNAlater (Sigma-Aldrich) and stored at −80°C until RNA extraction using 500 μl of TRIzol (Invitrogen). The RNA quality was evaluated using an Agilent 2100 Bioanalyzer (Agilent Technologies). The mean RNA integrity number (RIN) of the samples was 8.5 ± 0.7.

A total of 15 samples were used for RNA sequencing: RESPO+ (*n* = 5), RESPO– (*n* = 5), and no-MFD (controls, *n* = 5). Milk production records (content and yield) in controls, RESPO+ and RESPO– animals, after and before FO supplementation are summarized in Supplementary Table S1; particularly, the milk fat percentage variation ranged between 21.74%−29.68% and 7.77%−10.81% in the RESPO+ and RESPO– groups, respectively. With the aim of maximizing transcriptomic differences between goats with and without MFD, the latter (i.e., the 5 no-MFD) corresponded to control samples collected from RESPO+ goats before offering them the diet that induced MFD.

The RNA sequencing was conducted at Centro Nacional de Análisis Genómico (CNAG, Barcelona, Spain), where the TruSeq Stranded Total RNA Library Prep Kit (Illumina, San Diego, CA, USA) was used for library preparation. A NovaSeq™ 6000 Sequencing System (Illumina) was used to generate stranded paired-end reads of 51 bp.

### Alignment and quantification

RNA-Seq data files were aligned to the ARS1 goat reference genome (GCF_001704415.1_ARS1 from NCBI database) using STAR v. 2.7.6a (22). In addition to the default arguments for the alignment, we added the options “–outFilterType BySJout” to reduce spurious junctions, “–outWigStrand Stranded” to indicate that our RNA-Seq data was stranded, and the option “–quantMode TranscriptomeSAM” to the necessary output for the quantification with RSEM software (23). For the quantification with RSEM, we used the option “–paired-end” to indicate our data were paired-end and “–no-bam-output” to indicate that no bam output should be created. In addition, we used the options “–estimate-rspd” to estimate the start position of the distribution, “–calc-ci” to calculate 95% credibility intervals and posterior mean estimates, “–seed 12345” to set the seed for the random number of generators used in calculating posterior mean estimates and credibility intervals, and “-p 8” to fix the parallel environment. RSEM software can deal with mapping uncertainty due to multi-mapped reads using the expectation maximization (EM) algorithm to estimate the maximum likelihood value of gene or transcript abundance (24).

**Figure 1:**
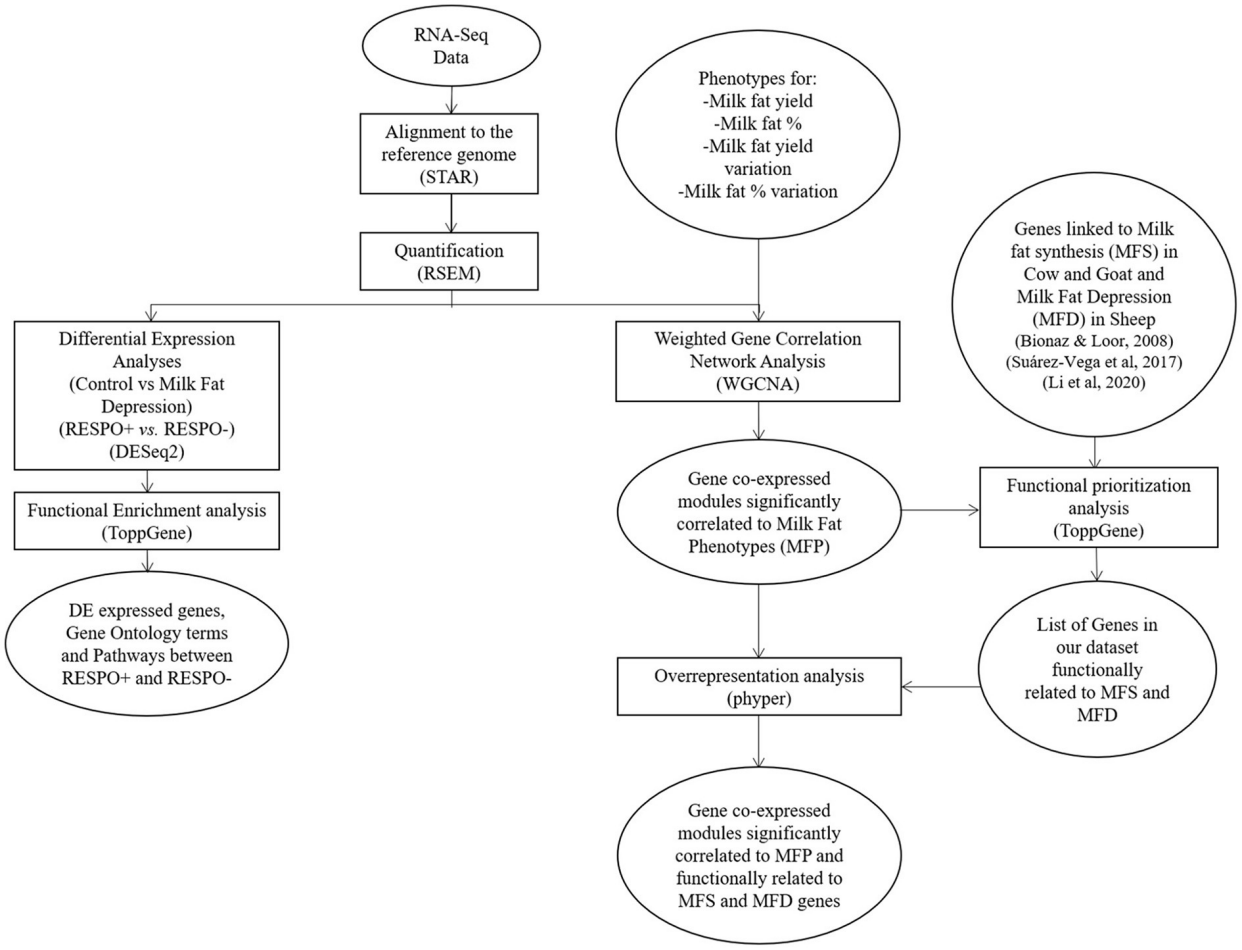
Bioinformatics workflow followed for the analysis of the RNA-Seq data.

After alignment and quantification, we followed two different analytical approaches: a standard DEA and a WGCNA. The bioinformatics workflow applied to analyze the RNA-Seq data is summarized in Figure 1.

### Differential gene expression analyses

We used the R package tximport (25) to import the results from the quantification step with RSEM software into R environment. The program DESeq2 v.1.34.0 (16) was used to perform the DEA. First, we used the function “collapseReplicates” in DESeq2 to collapse technical replicates. We computed the Euclidean distance among the samples using “dist” function in R and plotted the hierarchical clustering using “hclust” to check for outliers. Moreover, PCAtools (26) was used to perform a principal component analysis (PCA) to cluster samples based on gene expression data. Then, we performed differential expression analysis to identify differentially expressed genes (DEG) between control (*n* = 5) and MFD samples [the latter including both RESPO+ (*n* = 5) and RESPO– samples (*n* = 4; after removing outliers)]. In addition, we performed a DEA to evaluate differences between RESPO+ (*n* = 5) and RESPO– goats (*n* = 4). For both analyses, we considered genes as differentially expressed at a *P*-adjusted value (*P*_adj_) <0.05, i.e., after the correction of *P*-values for multiple testing using the Benjamini–Hochberg's approach.

### Functional enrichment analyses

Functional enrichment analyses were performed using the web-tool ToppFun in ToppGene Suite (27). Functional enrichment was performed for Gene Ontology (GO):Molecular function, GO:Biological Processes, GO:Cellular Component, and Pathway databases. The following options were applied to perform the analyses: “*P*-value Method = probability density function,” “multiple test correction = false discovery rate (FDR),” “FDR cutoff < 0.05,” “Gene Limits 1 ≤ *n* ≤ 2,000.” These analyses were performed for the different lists of DEGs obtained from the previous analyses, i.e., the upregulated or downregulated genes identified by the different comparisons between control and MFD samples and between RESPO+ and RESPO– samples.

### Weighted gene correlation network analyses

Expression values of genes were normalized using DESeq2 to obtain a matrix of counts to be used in the R package WGCNA (18), for weighted gene co-expression network construction. Briefly, the similarity matrix between each pair of genes across all samples was constructed by calculating Pearson correlations. Then, the similarity matrix was transformed into an adjacency matrix (*A*) raised to a soft threshold power based on the free-scale topology criterion. We used the function “pickSoftThreshold” to calculate the soft-thresholding power. In this study, the soft threshold (β parameter) was 12, which corresponds to a free-scale topology of *R*^2^ > 0.80. Subsequently, the topological overlap matrix (TOM) was used to define modules based on dissimilarity (1-TOM). Then, a hierarchical clustering from the dissimilarity matrix was generated with the “hclust” function. Modules of co-expression were then detected by using the dynamic tree cut (DTC) algorithm. The minimum size selected to construct the gene co-expression modules was 15.

Once the gene modules were detected, their associations with the studied milk fat traits (fat yield, fat percentage, variation in fat yield, and variation in fat percentage; Supplementary Table S1) were estimated using the correlation between the module eigengene (ME) and phenotypic values for each trait and animal, allowing the identification of modules highly correlated with the interest traits. Genes of modules with significant module-trait associations (*P*-value ≤ 0.05), for at least one trait, were used for functional enrichment analysis.

### Functional enrichment analysis for milk fat genes

To determine if the gene co-expressed modules significantly correlated to the phenotypic traits were functionally enriched in genes involved in milk fat synthesis or MFD, we developed the following functional enrichment analysis. First, we performed a functional prioritization analysis on ToppGene (27) using the genes from the gene modules significantly correlated to our traits as the test list. The training set was a list of 432 known genes linked to milk fat synthesis in cattle and goats, and to MFD in sheep (Supplementary Table S2) (11, 28, 29). The functional enrichment analysis for the test set was performed for the Gene Ontology (GO) (GO:Biological Process, GO:Molecular Function, and GO:Cellular Component), and the Pathway and Gene family databases, using as *P*-value method the probability density function and FDR < 0.05. Genes in the test set were considered functionally prioritized when the overall *P*-value for the analysis was <0.05.

Once we had our list of genes functionally associated to milk fat synthesis and/or MFD, composed by the training gene set and the functionally prioritized genes (milk fat gene list, 850 genes; Supplementary Table S3), we performed an overrepresentation analysis to check which of the gene co-expressed modules was enriched for the genes in this list. A hypergeometric test was performed for each significantly correlated module of genes using the phyper package (30) in R. For each evaluated module, we computed the probability that genes overlapping (specific for each gene co-expressed module) with the milk fat gene list was higher than expected by chance (i.e., overrepresented) using as background the 11,624 genes used to create the weighted-gene correlation networks. The *P*-value obtained was corrected for multiple testing using FDR and Bonferroni methods with the *P*.adjust function in R.

## Results

### Sequencing and alignment of the goat MSCs transcriptome

The statistics regarding RNA-Seq are summarized in Supplementary Table S4. The average of reads per sample was 37,335,520 (SD = 4,929,042.77), with a mode of technical replicates per sample of 2. The mapping rate to the goat reference genome *Capra hircus* assembly ARS1 was 93.86 % (SD = 0.01), with a percentage of uniquely mapped reads of 74.87% (SD = 0.05) and 19% of multi-mapping reads (SD = 0.04).

### Gene expression levels and sample distribution

Gene counts normalized using the Fragments per Kilobase per Million Mapped Reads (**FPKM**) method were used to evaluate gene expression levels. According to their expression, genes were classified into four different categories: genes with an expression lower than 0.1 FPKMs, which were not used in subsequent analyses, low expressed (<10 FPKMs), middle expressed (>10–500 FPKMs), and highly expressed (>500 FPKMs) genes (Supplementary Figure S1). The majority of genes ranged between 10 and 500 FPKM. Among 66 most expressed genes (>500 FPKMs) in goats, the top expressed genes were caseins (*CSN1S1, CSN1S2, CSN2*, and *CSN3*) and whey (*PAEP* and *LALBA*) proteins.

**Figure 2:**
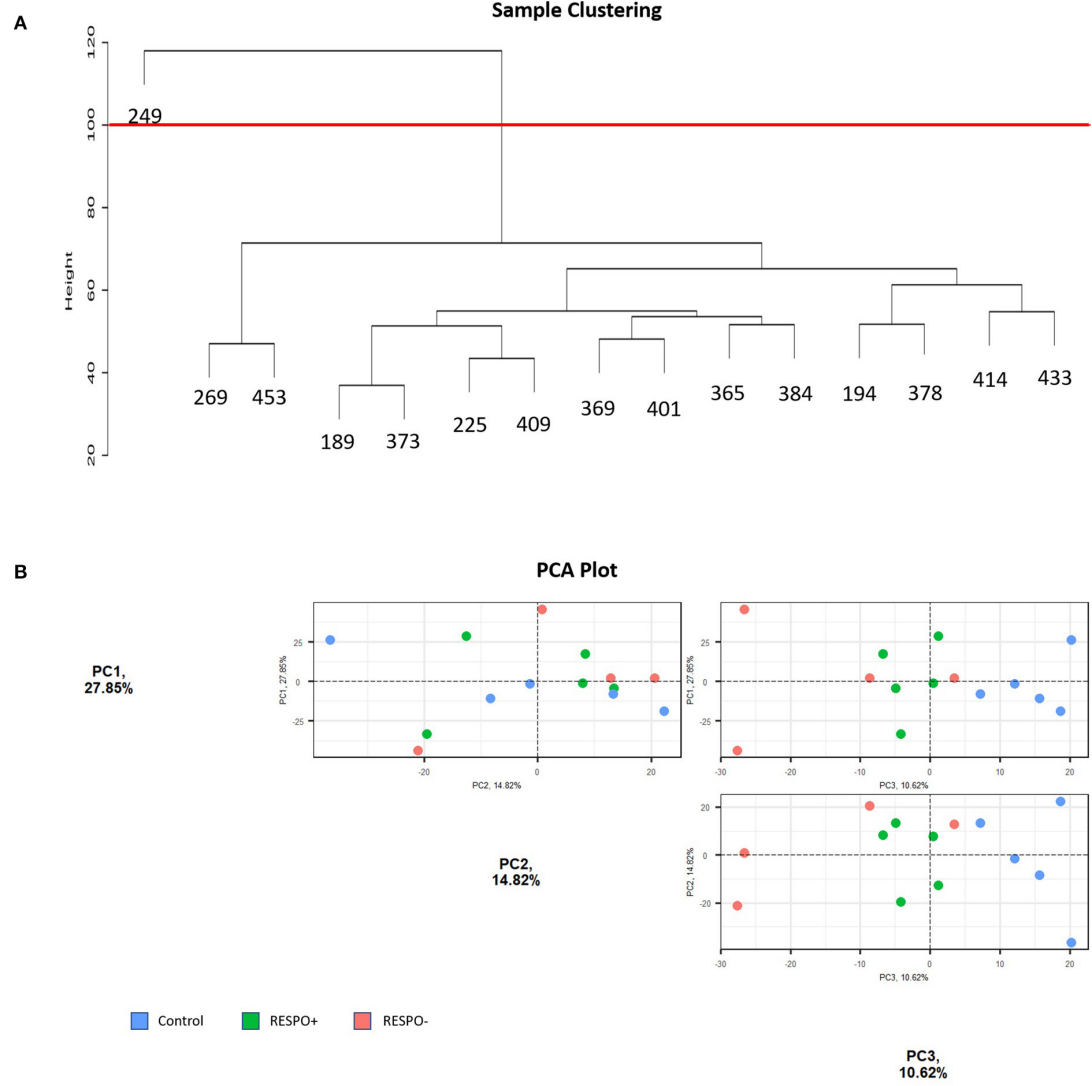
Sample Distribution of the RNA-Seq data. **(A)** Hierarchical clustering of samples based on Euclidean distance. The red line represents the threshold fixed to discard outliers **(B)** Principal Component Analysis (PCA) plots for PC1, PC2, and PC3 of milk somatic cell transcriptomes from control (blue) and more (RESPO+; green) or less (RESPO–; red) responsive goats to a diet inducing milk fat depression (MFD).

Based on the Euclidean distance, we removed one outlier (ID = RESPO2_249 sample) from the RNA-Seq dataset (Figure 2A). Regarding the PCA, Figure 2B shows the three principal components accounting for more than 10% of the variance. Altogether, PC1, PC2, and PC3 explained 53.29% of the variance. It was expected that lipid supplementation would not explain a high percentage of the variance in the PCA analysis when evaluating the whole transcriptome because of the animal variability and the fact that the number of genes expected to have a high impact on the complex quantitative traits is low. The first principal component (PC1), accounting for 27.85% of the variance, and the PC2 (accounting for 14.82%) were not able to group the samples by the conditions (Figure 2B). Nevertheless, the PC3, accounting for 10.62% of the variance, better discriminates the different conditions evaluated.

### Differentially expressed genes between control and MFD

A total of 45 genes were differentially expressed (*P*_adj_ < 0.05) between control and MFD goats (Supplementary Table S5). Thirty-three of these were upregulated in goats with MFD and 12 in the control. The top three differentially expressed genes were a ncRNA *LOC102181690* (*P*_adj_ = 1.16E−14, log_2_FoldChange= −2.16) and the gene coding for 40S ribosomal protein S27 (*LOC102171808*, *P*_adj_ = 0.0004, log_2_FoldChange= −2), both upregulated in MFD samples, and the *SREBF1-like* (*LOC108635517*, *P*_adj_ = 3.88E−05, log_2_FoldChange = 1.11), upregulated in control samples.

After functional enrichment analyses, genes upregulated in the goats with MFD were significantly clustered [False Discovery Rate Benjamini and Hochberg (FDR B&H) <0.05] in seven GO terms for the Molecular function category (GO:Molecular function), 50 terms in the GO:Biological Process category, 15 terms in the GO:Cellular Component category, and 37 in the pathway databases (Supplementary Table S6). The highest enriched GO terms for each category were “structural constituent of ribosome” (seven genes, FDR B&H = 3.966E−8) for GO:Molecular function, “SRP-dependent cotranslational protein targeting to membrane,” “cotranslational protein targeting to membrane,” “nuclear-transcribed mRNA catabolic process, nonsense-mediated decay,” “protein targeting to ER” and “establishment of protein localization to endoplasmic reticulum” (six genes, FDR B&H = 1.985E−7) for GO:Biological Process and “ribosomal subunit” for GO:Cellular Component (seven genes, FDR B&H = 7.833E−8). The highest enriched pathway was “Selenocysteine synthesis” (BioSystems: REACTOME, seven genes, FDR B&H = 2.052E−9).

Genes upregulated in control were significantly enriched in 30, 14, and 14 GO terms for the Molecular Function, Biological Process and Cellular Component categories, respectively, and 50 pathways (Supplementary Table S7). The top enriched GO terms for GO:Molecular function were “chromatin binding” and “transcription factor binding” (four genes, FDR B&H = 6.844E−3). For the GO:Biological Processes the highest enriched GO terms were “positive regulation of nucleic acid-templated transcription,” “positive regulation of transcription, DNA-templated,” and “positive regulation of RNA biosynthetic process” (six genes, FDR B&H = 1.801E−2). For the GO:Cellular Component the top enriched GO term was “Golgi trans cisterna” (two genes, FDR B&H = 1.166E−3), and for the pathway databases “Chromatin organization,” and “Chromatin modifying enzymes” (BioSystems: REACTOME,4 genes, FDR B&H = 1.609E−3).

### Differentially expressed genes between RESPO+ and RESPO–

There were 18 genes differentially expressed (*P*_adj_ < 0.05) between RESPO– and RESPO+ goats, 13 were upregulated in the RESPO+ animals and five in RESPO– (Supplementary Table S8). The top three differentially expressed genes were *DBI* (*P*_adj_ = 0.002, 0.56), *PBXIP1* (*P*_adj_ = 0.006, −0.89) and *ELMSAN1* (*P*_adj_ =0.006, −0.63). Results from the functional enrichment analyses for the upregulated genes in RESPO+ are detailed in Supplementary Table S9. The top enriched GO term among those significantly enriched was potassium channel inhibitor activity (two genes, FDR B&H = 1.323E−3) for the GO:Molecular function category (18 terms, FDR B&H < 0.05), regulation of potassium ion transmembrane transport (three genes, FDR B&H = 5.715E−3) for the GO:Biological Process category (50 terms, FDR B&H < 0.05), and anchoring junction (four genes, FDR B&H = 3.794E−2) for the GO:Cellular Component (16 terms, FDR B&H < 0.05). No pathways were significantly enriched.

All results for the functional enrichment analyses of genes upregulated in RESPO– are in Supplementary Table S10. For the five upregulated genes, 11 terms were enriched for the GO:Molecular function category; “oxidoreductase activity, acting on the CH-OH group of donors, quinone or similar compound as acceptor” and “glycerol-3-phosphate dehydrogenase (quinone) activity” (one gene, FDR B&H = 5.407E-3) being the highest enriched terms. For the GO: Biological Process category, we found 22 terms significantly enriched; “positive regulation of CoA-transferase activity,” “regulation of CoA-transferase activity,” “glycerol-3-phosphate catabolic process,” and “negative regulation of protein lipidation” (one gene, FDR B&H = 2.958E-2) being the top enriched ones. For the GO:Cellular component category, four terms were significantly enriched (FDR B&H < 0.05), with “glycerol-3-phosphate dehydrogenase complex” (one gene, FDR B&H = 1.361E−2) being the top enriched term. Moreover, we found 22 pathways significantly enriched, “Triglyceride Biosynthesis” (BioSystems: REACTOME, two genes, FDR B&H = 2.887E−3) being the top enriched pathway.

### Co-expressed gene modules and correlations with milk fat yield, milk fat percentage and milk fat variation traits

**Figure 3:**
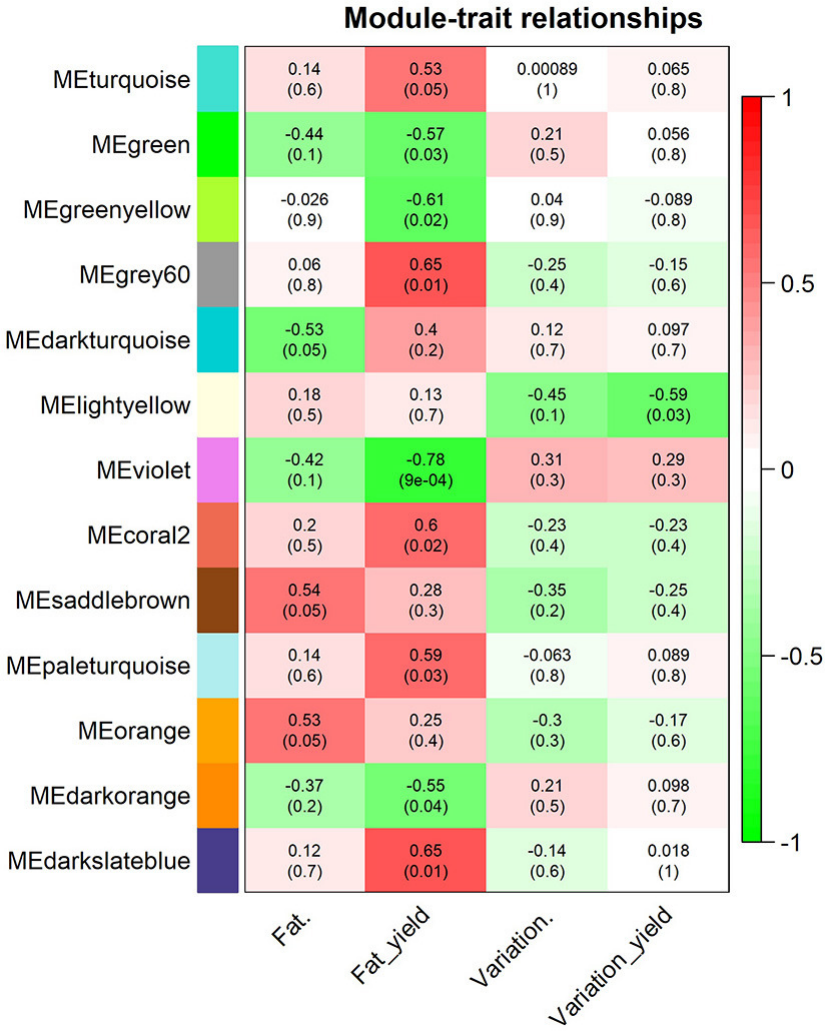
Significant module-trait associations found between the module eigengenes (ME) and the milk-fat traits analyzed [fat percentage (Fat.), fat yield (Fat_yield), variation in fat percentage (Variation.), and variation in fat yield (Variation_yield)]. Each row corresponds to a module eigengene labeled with colors and each column to a milk fat trait. Individual cells contain Pearson's correlation coefficients (outside parentheses) and the *P*-values of the correlation (within parentheses). The red to green color within the cells represents the positive (red) or negative (green) correlation of the modules with the milk fat traits.

Using the WGCNA approach, we identified 67 modules of co-expressed and highly interconnected genes, each module being assigned to different colors (Figure 3). Among the modules identified, three were significantly correlated to milk fat percentage (orange, saddlebrown, and darkturqouise), nine significantly correlated to milk fat yield (darkorange, green, violet, greenyellow, turquoise, paleturquoise, darkslateblue, grey60, and coral2) and 1 significantly correlated to the variation in milk fat yield due to MFD (lightyellow). The module with the highest correlation with any of the traits was the violet, with a correlation of −0.78 with milk-fat yield (*P*-value = 0.0009, 46 genes). The unique module associated with milk fat yield variation due to MFD was lightyellow with a correlation of −0.59 (*P*-value = 0.025, 95 genes). None of the modules was significant for all traits together. The list of all modules and associated genes is provided in Supplementary Table S11.

The functional enrichment analyses performed with the training list of genes (Supplementary Table S2) confirmed their relevant function in processes related to milk fat synthesis. The highest enriched terms for the GO:Molecular function, GO:Biological Processes, GO:Cellular Component, Pathway and Gene family categories were “acyltransferase activity” (31 genes, FDR B&H = 4.255E−11), “lipid metabolic process” (130 genes, FDR B&H = 5.813E−46), endoplasmic reticulum subcompartment (64 genes, FDR B&H = 3.769E−11), Metabolism of lipids and lipoproteins (BioSystems:REACTOME, 103 genes, FDR B&H = 3.769E−11), and Acyl-CoA dehydrogenase family (six genes, FDR B&H = 7.984E−7), respectively. After the functional prioritization analyses, 106 genes from the test gene set (genes from modules significantly correlated to milk-fat phenotypes) overlapped with genes in the training set, and 745 genes were functionally prioritized (Supplementary Table S12). In total, 25% of the genes in our test set were functionally associated to milk fat synthesis or MFD.

**Figure 4:**
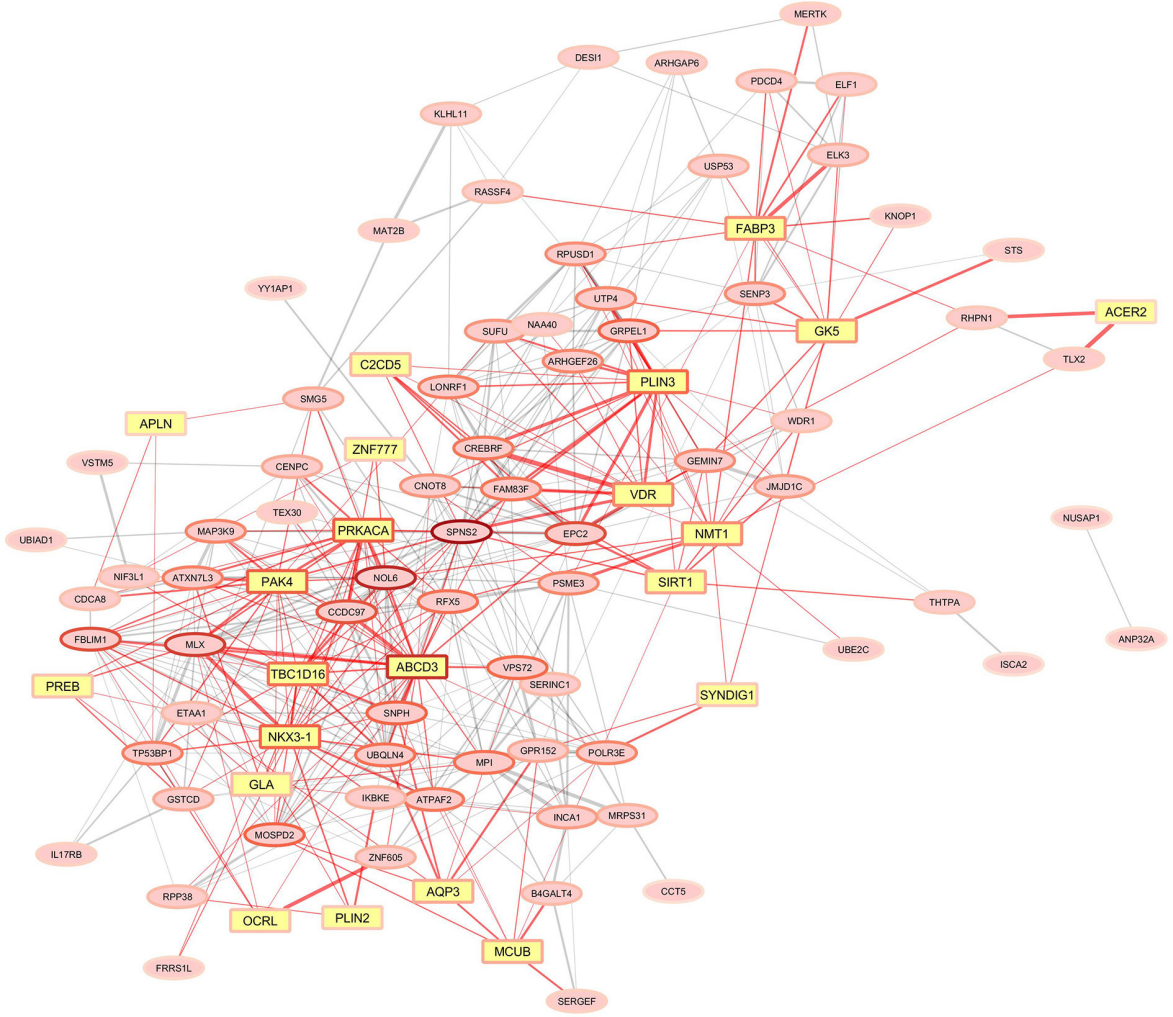
Gene co-expression network for the lightyellow module significantly correlated with milk fat-yield reduction due to the MFD-inducing diet. Yellow squared nodes (yellow genes) represent genes functionally associated with mammary lipid metabolism in ruminants. Red edges indicate the connections of yellow genes in the network. The width of the edges corresponds to the weight in the WGCNA. The border color of the node is related to the number of connections, reddish nodes represent highly connected hub genes, and lighter red border colors of nodes represent genes with fewer connections.

Once we had the milk fat gene list (Supplementary Table S3), we performed the overrepresentation analyses for the gene modules significantly correlated to the milk-fat phenotypes. The results are summarized in Table 1. There were 10 modules overrepresented; the turquoise one was the highest enriched. The lightyellow module, was the only one significantly correlated to the variation in fat yield due to MFD, and was also enriched with 21 genes functionally related to milk fat synthesis (Figure 4).

**Table 1:** Results from the overrepresentation analyses among genes in modules significantly correlated to milk fat phenotypes and the milk fat gene list^a^.

**Module name**	**Number of genes in the module**	**Common genes (modules vs. milk fat gene list)**		***P*-value**	**FDR B&H**	**Bonferroni**
		**Number**	**%**			
Turquoise	2,014	564	28.00	1.40E−246	1.82E−245	1.82E−245
Green	552	114	20.65	5.77E−26	3.75E−25	7.50E−25
Greenyellow	210	50	23.81	8.96E−15	3.88E−14	1.16E−13
Grey60	110	24	21.82	2.56E−07	8.32E−07	3.33E−06
Darkturquoise	83	20	24.10	3.45E−07	8.98E−07	4.49E−06
Lightyellow	95	21	22.11	9.48E−07	2.05E−06	1.23E−05
Violet	46	12	26.09	1.64E−05	3.05E−05	0.0002
Coral2	23	6	26.09	0.0009	0.0015	0.0123
Saddlebrown	53	10	18.87	0.0013	0.0019	0.0173
Paleturquoise	47	9	19.15	0.0018	0.0023	0.0232
Orange	65	8	12.31	0.0459	0.0497	0.5968
Darkorange	64	8	12.50	0.0422	0.0497	0.5482
Darkslateblue	30	4	13.33	0.0641	0.0641	0.8338

a The milk fat gene list (obtained from the literature and prioritization analyses) is detailed in Supplementary Table S3.

## Discussion

We investigated changes in the milk somatic cell transcriptome in goats subjected to a diet known to induce MFD through two RNA-Seq analytical approaches to determine genes and gene networks involved in individual differences in the degree of MFD. First, the DEA allowed us to determine those genes differentially expressed between the analyzed conditions (control vs. MFD and RESPO– vs. RESPO+). Second, the WGCNA allowed us to gain insights into the genetic signatures related to complex quantitative phenotypes such as milk fat synthesis and individual MFD susceptibility. Although DEA is the methodology commonly chosen to discriminate DEGs between different experimental conditions, this approach can have several limitations. In addition to the fact that genes are studied individually, the application of this approach can be challenging when molecular differences between the analyzed conditions are small (31). On the other hand, the WGCNA constructs networks of genes based on the pairwise correlations between their expression (18) such that genes contributing to the same biological process are usually under the same transcriptional regulation and tend to have a correlated expression performance (32). Thus, it has been shown that the combination of DEA and WGCNA approaches can be advantageous for identifying genes involved in functional differences between similar tissues where gene expression levels do not differ much (31). The complementarity of these two methodologies helped us gain insights into the genetic basis of a quantitative phenotype, diet-induced MFD in the lactating mammary gland, where most reads (~90%) account for major proteins involved in milk synthesis, and only subtle differences in gene expression exist across animals (absolute log_2_FoldChange hardly surpass 2).

In DEA, a total of 45 genes exhibited differences in expression. Among them, *SREBF1-like* and *SREBF* genes were upregulated in the control group. Sterol regulatory element-binding transcription factors (SREBFs) act as main regulators in controlling enzymes involved in de novo synthesis of fatty acids and cholesterol (33, 34). Our findings agree with reports in cattle and sheep, where decreases in *SREBF1* expression have been associated with MFD (5, 35, 36). Although a recent study in goats under starch-induced MFD found no variation in *SREBF* abundance, a decrease in *INSIG1* expression was detected, which may affect SREBP activity (15). Similarly, a previous study performed by our research group, aiming to identify differentially expressed genes from the MSCs transcriptome of FO-induced MFD in dairy sheep, did not detect differential expression for *SREBF1*, but the *SREBP signaling pathway* was enriched (11). Among the GO (Molecular Function and Biological Processes) terms and biological pathways that were enriched in the present study, several contained genes involved in the regulation of RNA transcription (*SREBF1, HIVEP3, ZNF827, PPRC1, DOT1L*, and *ASH1L*), chromatin-modifying enzymes (*NCOR2, CHD4, DOT1L*, and *ASH1L*), and histone methyltransferase activity (*DOT1L* and *ASH1L*).

Dietary lipids have been postulated as regulators of gene expression in a hormonal-independent manner through regulation of activity and/or abundance of transcription factors and nuclear receptors affecting the transcription of many genes (37, 38). Moreover, epigenetic changes in gene expression due to modifications in the chromatin have been attributed to dietary unsaturated fatty acids in non-ruminant animal models (39). For upregulated genes during MFD, we mainly observed genes encoding proteins that are structural constituents of the ribosome (*MRPL27, RPL24, RPLP0, RPL3, RPS7, RPL13*, and *RPS8*), supporting the hypothesis that *RPL* genes are targets of dietary long-chain polyunsaturated fatty acids in rodents (40). On the contrary, these results contrast with the previously reported stability of *RPLP0* expression in the mammary gland of ruminants (41), which prompted its use as a reference gene in nutritional trials, including the study of mammary lipogenesis in goats under marine lipid-induced MFD conditions (42).

Once the main DEGs due to the MFD-inducing diet were determined, we identified those differentially expressed in RESPO+ and RESPO– goats. Only 18 genes were deemed differentially expressed. The upregulated GO terms clustering genes upregulated in RESPO+ goats were mainly related to the regulation of potassium ion transmembrane transport (*WNK4, BIN1*, and *CAV1*), specifically, potassium channel inhibitor activity. Supplementation with potassium-based products has been suggested to improve milk fat concentration in dairy ruminants. However, this effect has not been related to a direct effect in the mammary gland, but to decreased production of ruminal *trans*-10 18:1 and *trans*-10*, cis*-12 CLA (43). In the companion study (8), FO supplementation tended to induce a greater increase in milk *trans*-10 18:1 concentration in RESPO+ than RESPO–, but no relationship between milk *trans*-10*, cis*-12 CLA and responsiveness to the MFD-inducing diet was detected.

In RESPO– goats, two upregulated genes (*GPD1* and *DBI*) explained the enrichment in the pathway “Triglyceride biosynthesis,” suggesting that less susceptible goats had higher synthesis of triglycerides in mammary cells. The *GPD1* encodes for glycerol-3-phosphate dehydrogenase 1, which acts in the reversible conversion of dihydroxyacetone phosphate (DHAP) and reduces nicotine adenine dinucleotide (NADH) to glycerol-3-phosphate (G3P) and NAD+, having a critical role in carbohydrate and lipid metabolism. A study in dairy cows and goats fed diets supplemented with starch, plant oil, or fish oil showed no differences in the expression of *GDP1* in the mammary gland due to changes in milk fat content in response to diets (44). This agrees with our findings when comparing control vs. MFD. However, the *GDP1* gene was differentially expressed when we compared RESPO+ and RESPO–, suggesting a relevant role of this gene in the individual differences in MFD severity.

Bernard et al. (44) reported a negative correlation between *GDP1* gene expression and the milk content of *cis*-11 16:1, a fatty acid that is present in FO. Although there is no research deciphering the biological meaning of this correlation, milk concentrations of *cis*-11 16:1 seem associated with reduced milk fat content (g/kg) (44–46). However, in the companion paper by Della Badia et al. (8), we observed a higher *cis*-11 16:1 proportion in RESPO– goats (*P* = 0.028), which would not support the negative correlation between *GDP1* gene and this fatty acid. In any event, further research is necessary to elucidate the mechanisms underlying these links and to verify whether they denote causation (e.g., a biological effect of *cis*-11 16:1 on gene expression) or not (i.e., a simple reflection of *cis*-11 16:1 supply with FO).

The other gene upregulated in RESPO– animals and linked to lipid metabolism was *DBI*. This gene, also known as *ACBP*, encodes for acyl-CoA binding protein, which plays a role in modulating the regulatory functions and utilization of long-chain fatty acyl-CoA, preventing the inhibitory effects of these compounds on enzyme activity (e.g., on mitochondrial acyl-CoA synthetase and acetyl-Coa carboxylase) (47). The *DBI* gene was downregulated in dairy cows and sheep displaying MFD due to the consumption of diets rich in unsaturated fatty acids (11, 35). In this study, *DBI* was not among the differentially expressed genes when comparing control vs. MFD groups, but when evaluating more and less susceptible animals (i.e., RESPO+ vs. RESPO–). Thus, it could be speculated that changes in the expression of *DBI* gene might represent a mechanism that protected mammary cells of RESPO– goats from excessive accumulation of long-chain fatty acyl-CoA due to FO supplementation (47).

In the WGCNA, most modules significantly correlated with milk fat phenotypes were related to milk fat yield, which would suggest that transcriptomic changes associated to milk fat decrease are subtle in comparison with those observed for milk fat synthesis. Our companion study postulated that some pre-existing variations in some traits, such as milk fat yield, may influence the susceptibility of sheep to dietary components inducing MFD, but this trend was not confirmed in goats (8). The relevance of this trait might explain that none of the modules correlated to milk fat yield was also correlated to variations in milk fat yield in response to diet, the latter variable having only one significantly correlated module. Regarding milk fat percentage traits, none of the gene modules correlated with milk fat percentage was correlated with milk fat yield. In small ruminants, milk fat yield and milk yield are usually positively correlated, whereas milk fat percentage is negatively correlated with milk yield, which has often been attributed to a dilution effect (48, 49). This fact supports the idea that different gene correlated networks might drive both traits. One of the genes included in the turquoise module, which positively correlated to milk fat yield, was *LALBA*. This gene encodes alpha-lactalbumin, an essential protein for lactose synthesis, a major milk osmolyte influencing milk volume (50, 51), which may account for the relationship between this module and milk fat yield. Although there were also some discrepancies in significant modules and their correlations to different phenotypic traits, nearly all modules were enriched in genes functionally related to milk fat synthesis, which would support their biological relevance in relation to the evaluated traits.

As the main objective of the work was to identify genes and gene networks related to the individual susceptibility of goats to MFD, we paid particular attention to the lightyellow module of co-expressed genes, which was negatively correlated to MFD. Among the 95 genes in this network, 21 functionally-related to milk fat traits were enriched (Figure 4). Within these, *FABP3* has been shown as the intracellular fatty acid transporter with the greatest expression in the lactating mammary gland of cows, together with the previously discussed *DBI*/*ACBP* (28). Moreover, *FABP3* has been demonstrated to be a target gene of *SREBP1* and *PPARG*, the central regulators of lipid metabolism (52). A study in cows demonstrated that oleic acid increases lipid droplet accumulation in mammary epithelial cells by affecting the expression of *FABP3* (53). In line with this, we detected within the lightyellow gene co-expressed network two members of perilipin family proteins (*PLIN2* and *PLIN3*) related to lipid droplets (54). Specifically, *PLIN2* is involved in mammary lipid droplet formation, stabilization and secretion in goat mammary epithelial cells and regulates triglyceride accumulation (55). Although further research is necessary, these findings suggest that alterations in fatty acid transport might play an important role in inter-animal differences in susceptibility to diet-induced MFD in goats.

## Conclusions

The combination of two approaches, DEA and WGCNA, helped elucidate MSC transcriptomic changes caused during diet-induced MFD in dairy goats. Results supported that MFD in dairy goats is mediated by *SREBF* downregulation and variations in additional transcription factors and chromatin-modifying enzymes. Regarding individual susceptibility to MFD, we uncovered several DEGs between RESPO+ and RESPO– goats (e.g., *DBI* and *GPD1*) and a co-related gene network linked to the decrease in milk fat. Although all candidate genes potentially related to individual susceptibility to MFD severity should be further investigated, alterations in fatty acid transport seem to play an important role.

## Data availability statement

The datasets presented in this study can be found in online repositories. The names of the repository/repositories and accession number(s) can be found at: https://www.ebi.ac.uk/biostudies/arrayexpress/studies/E-MTAB-11581?accession=E-MTAB-11581.

## Ethics statement

The animal study was reviewed and approved by Research Ethics Committees of the Instituto de Ganadería de Montaña, the Spanish National Research Council (CSIC), and the Junta de Castilla y León (Spain).

## Author contributions

GH, PF, and PT designed the study with the cooperation of J-JA. GH, PF, and PT coordinated all tasks involved in animal maintenance, diet design and phenotype recording. PT did RNA extractions. AS-V carried out the bioinformatic analyses. AS-V and BG-G wrote the first draft of the paper with the cooperation of PT, PF, J-JA, and JL. All authors read and approved the content of the paper.

## References

[1] Doreau M Bauchart D Chilliard Y. Enhancing fatty acid composition of milk and meat through animal feeding1. Anim Prod Sci. (2010) 51:19–29. 10.1071/AN1004324576480

[2] Shingfield KJ Bonnet M Scollan ND. Recent developments in altering the fatty acid composition of ruminant-derived foods. Animal. (2013) 7:132–62. 10.1017/S1751731112001681 23031638

[3] Bauman DE Tyburczy C O'Donnell AM Lock AL. Milklipids: Conjugated linoleic acid. In:Fuquay JM Fox PF McSweeney PLH, editors. Encyclopedia of Dairy Sciences. 2nd ed. San Diego, CA: Academic Press (2011). p. 660–4.

[4] Salter AM. Dietary fatty acids and cardiovascular disease. Animal. (2013) 7:163–71. 10.1017/S1751731111002023 23031737

[5] Carreño D Hervás G Toral PG Castro-Carrera T Frutos P. Fish oil-induced milk fat depression and associated downregulation of mammary lipogenic genes in dairy ewes. J Dairy Sci. (2016) 99:7971–81. 10.3168/jds.2016-11019 27474983

[6] Reynolds CK Cannon VL Loerch SC. Effects of forage source and supplementation with soybean and marine algal oil on milk fatty acid composition of ewes. Anim Feed Sci Technol. (2006) 131:333–57. 10.1016/j.anifeedsci.2006.06.015

[7] Weimer PJ Stevenson DM Mertens DR. Shifts in bacterial community composition in the rumen of lactating dairy cows under milk fat-depressing conditions. J Dairy Sci. (2010) 93:265–78. 10.3168/jds.2009-2206 20059925

[8] Della Badia A Hervás G Toral PG Frutos P. Individual differences in responsiveness to diet-induced milk fat depression in dairy sheep and goats. J Dairy Sci. (2021) 104:11509–21. 10.3168/jds.2021-20414 34364637

[9] Müller M Kersten S. Nutrigenomics: goals and strategies. Nat Rev Genet. (2003) 4:315–22. 10.1038/nrg1047 12671662

[10] Osorio JS Vailati-Riboni M Palladino A Luo J Loor JJ. Application of nutrigenomics in small ruminants: Lactation, growth, and beyond. Small Rumin Res. (2017) 154:29–44. 10.1016/j.smallrumres.2017.06.021

[11] Suárez-Vega A Toral PG Gutiérrez-Gil B Hervás G Arranz JJ Frutos P. Elucidating fish oil-induced milk fat depression in dairy sheep: Milk somatic cell transcriptome analysis. Sci Rep. (2017) 7:45905. 10.1038/srep45905 28378756 PMC5381099

[12] Suárez-Vega A Gutiérrez-Gil B Toral PG Hervás G Arranz JJ Frutos P. Conjugated linoleic acid. (CLA)-induced milk fat depression: application of RNA-Seq technology to elucidate mammary gene regulation in dairy ewes. Sci Rep. (2019) 9:1–9. 10.1038/s41598-019-40881-3 30872673 PMC6418271

[13] Grajales SMB Zuluaga JJE Herrera AL Osorio NR Vergara DMB. RNA-seq differential gene expression analysis in mammary tissue from lactating dairy cows supplemented with sunflower oil. Anim Prod Sci. (2020) 60:758–71. 10.1071/AN19107

[14] Savoini G Zorini FO Farina G Agazzi A Cattaneo D Invernizzi G. Effects of fat supplementation in dairy goats on lipid metabolism and health status. Animals. (2019) 9:917. 10.3390/ani9110917 31689973 PMC6912558

[15] Zheng L Wu S Shen J Han X Jin C Chen X. High rumen degradable starch decreased goat milk fat via trans-10, cis-12 conjugated linoleic acid-mediated downregulation of lipogenesis genes, particularly, INSIG1. J Anim Sci Biotechnol. (2020) 11:1–14. 10.1186/s40104-020-00436-3 32280461 PMC7132897

[16] Love MI Huber W Anders S. Moderated estimation of fold change and dispersion for RNA-seq data with DESeq2. Genome Biol. (2014) 15:550. 10.1186/s13059-014-0550-8 25516281 PMC4302049

[17] Ritchie ME Phipson B Wu D Hu Y Law CW Shi W. limma powers differential expression analyses for RNA-sequencing and microarray studies. Nucleic Acids Res. (2015) 43:e47. 10.1093/nar/gkv007 25605792 PMC4402510

[18] Langfelder P Horvath S WGCNA. An R package for weighted correlation network analysis. BMC Bioinformatics. (2008) 9:1–13. 10.1186/1471-2105-9-559 19114008 PMC2631488

[19] Toral PG Hervás G Suárez-Vega A Arranz JJ Frutos P. Isolation of RNA from milk somatic cells as an alternative to biopsies of mammary tissue for nutrigenomic studies in dairy ewes. J Dairy Sci. (2016) 99:8461–71. 10.3168/jds.2016-11184 27497905

[20] Boutinaud M Jammes H. Potential uses of milk epithelial cells: a review. Reprod Nutr Dev. (2002) 42:133–47. 10.1051/rnd:2002013 12216959

[21] Canovas A Rincon G Bevilacqua C Islas-Trejo A Brenaut P Hovey RC. Comparison of five different RNA sources to examine the lactating bovine mammary gland transcriptome using RNA-sequencing. Sci Rep. (2014) 4:5297. 10.1038/srep05297 25001089 PMC5381611

[22] Dobin A Davis CA Schlesinger F Drenkow J Zaleski C Jha S. ultrafast universal RNA-seq aligner. Bioinformatics. (2013) 29:15–21. 10.1093/bioinformatics/bts635 23104886 PMC3530905

[23] Li B Dewey CN RSEM. accurate transcript quantification from RNA-Seq data with or without a reference genome. BMC Bioinformatics. (2011) 12:323. 10.1186/1471-2105-12-323 21816040 PMC3163565

[24] Deschamps-Francoeur G Simoneau J Scott MS. Handling multi-mapped reads in RNA-seq. Comput Struct Biotechnol J. (2020) 18:1569–76. 10.1016/j.csbj.2020.06.014 32637053 PMC7330433

[25] Soneson C Love MI Robinson MD. Differential analyses for RNA-seq: transcript-level estimates improve gene-level inferences. F1000Res. (2016) 4:1521. 10.12688/f1000research.7563.2 26925227 PMC4712774

[26] Blighe K Lun A. PCAtools: everything Principal Components Analysis. (2019). Available online at: https://github.com/kevinblighe/PCAtools (accessed December 23, 2021).

[27] Chen J Bardes EE Aronow BJ Jegga AG. ToppGene Suite for gene list enrichment analysis and candidate gene prioritization. Nucleic Acids Res. (2009) 37:W305–11. 10.1093/nar/gkp427 19465376 PMC2703978

[28] Bionaz M Loor JJ. Gene networks driving bovine milk fat synthesis during the lactation cycle. BMC Genomics. (2008) 9:366. 10.1186/1471-2164-9-366 18671863 PMC2547860

[29] Li C Zhu J Shi H Luo J Zhao W Shi H. Comprehensive transcriptome profiling of dairy goat mammary gland identifies genes and networks crucial for lactation and fatty acid metabolism. Front Genet. (2020) 11:878. 10.3389/fgene.2020.00878 33101357 PMC7545057

[30] Federico A Monti S. hypeR: an R package for geneset enrichment workflows. Bioinformatics. (2020) 36:1307–8. 10.1093/bioinformatics/btz700 31498385 PMC7998712

[31] Abbassi-Daloii T Kan HE Raz V 't Hoen PAC. Recommendations for the analysis of gene expression data to identify intrinsic differences between similar tissues. Genomics. (2020) 112:3157–65. 10.1016/j.ygeno.2020.05.026 32479991

[32] de la Fuente A. From ‘differential expression' to ‘differential networking' – identification of dysfunctional regulatory networks in diseases. Trends Genet. (2010) 26:326–333. 10.1016/j.tig.2010.05.001 20570387

[33] Horton JD Shah NA Warrington JA Anderson NN Park SW Brown MS. Combined analysis of oligonucleotide microarray data from transgenic and knockout mice identifies direct SREBP target genes. Proc Natl Acad Sci U S A. (2003) 100:12027–32. 10.1073/pnas.1534923100 14512514 PMC218707

[34] Rudolph MC Monks J Burns V Phistry M Marians R Foote MR. Sterol regulatory element binding protein and dietary lipid regulation of fatty acid synthesis in the mammary epithelium. Am J Physiol Endocrinol Metab. (2010) 299:E918–27. 10.1152/ajpendo.00376.2010 20739508 PMC3006251

[35] Ibeagha-Awemu EM Li R Ammah AA Dudemaine PL Bissonnette N Benchaar C. Transcriptome adaptation of the bovine mammary gland to diets rich in unsaturated fatty acids shows greater impact of linseed oil over safflower oil on gene expression and metabolic pathways. BMC Genomics. (2016) 17:104. 10.1186/s12864-016-2423-x 26861594 PMC4748538

[36] Chen W Alexandre PA Ribeiro G Fukumasu H Sun W Reverter A Li Y. Identification of predictor genes for feed efficiency in beef cattle by applying machine learning methods to multi-tissue transcriptome data. Front Genet. (2021) 12:619857. 10.3389/fgene.2021.619857 33664767 PMC7921797

[37] Jump DB. Dietary polyunsaturated fatty acids and regulation of gene transcription. Curr Opin Lipidol. (2002) 13:155–64. 10.1097/00041433-200204000-00007 11891418

[38] Sampath H Ntambi JM. Polyunsaturated fatty acid regulation of gene expression. Nutr Rev. (2004) 62:333–9. 10.1111/j.1753-4887.2004.tb00058.x 15497766

[39] Abbas A Witte T Patterson WL Fahrmann JF Guo K Hur J. Epigenetic reprogramming mediated by maternal diet rich in omega-3 fatty acids protects from breast cancer development in F1 offspring. Front Cell Dev Biol. (2021) 9:1517. 10.3389/fcell.2021.682593 34179012 PMC8222782

[40] Berger A Roberts MA. Unraveling Lipid Metabolism with Microarrays. Bosa Roca, FL: CRC Press. (2004). 445 p.

[41] Bonnet M Bernard L Bes S Leroux C. Selection of reference genes for quantitative real-time PCR normalisation in adipose tissue, muscle, liver and mammary gland from ruminants. Animal. (2013) 7:1344–53. 10.1017/S1751731113000475 23552195

[42] Fougère H Bernard L. Effect of diets supplemented with starch and corn oil, marine algae, or hydrogenated palm oil on mammary lipogenic gene expression in cows and goats: a comparative study. J Dairy Sci. (2019) 102:768–79. 10.3168/jds.2018-15288 30343921

[43] Jenkins TC Bridges WC Harrison JH Young KM. Addition of potassium carbonate to continuous cultures of mixed ruminal bacteria shifts volatile fatty acids and daily production of biohydrogenation intermediates. J Dairy Sci. (2014) 97:975–84. 10.3168/jds.2013-7164 24359822

[44] Bernard L Toral PG Chilliard Y. Comparison of mammary lipid metabolism in dairy cows and goats fed diets supplemented with starch, plant oil, or fish oil. J Dairy Sci. (2017) 100:9338–51. 10.3168/jds.2017-12789 28888611

[45] Bernard L Leroux C Rouel J Delavaud C Shingfield KJ Chilliard Y. Effect of extruded linseeds alone or in combination with fish oil on intake, milk production, plasma metabolite concentrations and milk fatty acid composition in lactating goats. Animal. (2015) 9:810–21. 10.1017/S1751731114003048 25491438

[46] Toral PG Hervás G Carreño D Belenguer A Frutos P. Comparison of milk fatty acid responses during fish oil- and trans-10 cis-12 18:2-induced milk fat depression in dairy ewes. Anim Feed Sci Technol. (2015) 210:66–73. 10.1016/j.anifeedsci.2015.09.024

[47] Alquier T Christian-Hinman CA Alfonso J Færgeman NJ. From benzodiazepines to fatty acids and beyond: revisiting the role of ACBP/DBI. Trends Endocrinol Metab. (2021) 32:890–903. 10.1016/j.tem.2021.08.009 34565656 PMC8785413

[48] Analla M Jiménez-Gamero I Muñoz-Serrano A Serradilla JM Falagán A. Estimation of genetic parameters for milk yield and fat and protein contents of milk from murciano-granadina goats. J Dairy Sci. (1996) 79:1895–8. 10.3168/jds.S0022-0302(96)76558-X 8923261

[49] Sánchez-Mayor M Pong-Wong R Gutiérrez-Gil B Garzón A de la Fuente LF Arranz JJ. Phenotypic and genetic parameter estimates of cheese-making traits and their relationships with milk production, composition and functional traits in Spanish Assaf sheep. Livest Sci. (2019) 228:76–83. 10.1016/j.livsci.2019.08.004

[50] Stinnakre MG Vilotte JL Soulier S Mercier JC. Creation and phenotypic analysis of alpha-lactalbumin-deficient mice. Proc Natl Acad Sci. (1994) 91:6544–8. 10.1073/pnas.91.14.6544 8022817 PMC44239

[51] Garcia-Gamez E Gutierrez-Gil B Sahana G Sanchez JP Bayon Y Arranz JJ. analysis for milk production traits in dairy sheep and genetic support for a QTN influencing milk protein percentage in the LALBA gene. PLoS ONE. (2012) 7:e47782. 10.1371/journal.pone.0047782 23094085 PMC3475704

[52] Ma L Corl BA. Transcriptional regulation of lipid synthesis in bovine mammary epithelial cells by sterol regulatory element binding protein-1. J Dairy Sci. (2012) 95:3743–55. 10.3168/jds.2011-5083 22720931

[53] Liang MY Hou XM Qu B Zhang N Li N Cui YJ Li QZ. Functional analysis of FABP3 in the milk fat synthesis signaling pathway of dairy cow mammary epithelial cells. In Vitro Cell Dev Biol Anim. (2014) 50:865–73. 10.1007/s11626-014-9780-z 24947174

[54] Sztalryd C Brasaemle DL. The perilipin family of lipid droplet proteins: gatekeepers of intracellular lipolysis. Biochim Biophys Acta Mol Cell Biol Lipids. (2017) 1862:1221–32. 10.1016/j.bbalip.2017.07.009 28754637 PMC5595658

[55] Shi H Zhu J Luo J Cao W Shi H Yao D. Genes regulating lipid and protein metabolism are highly expressed in mammary gland of lactating dairy goats. Funct Integr Genomics. (2015) 15:309–21. 10.1007/s10142-014-0420-1 25433708

