# Supplementary figures and images for: Elucidating genes and gene networks linked to individual susceptibility to milk fat depression in dairy goats
Source: Front Vet Sci. 2022 Dec 15;9:1037764. doi: 10.3389/fvets.2022.1037764 (PMC9798324; doi:10.3389/fvets.2022.1037764)

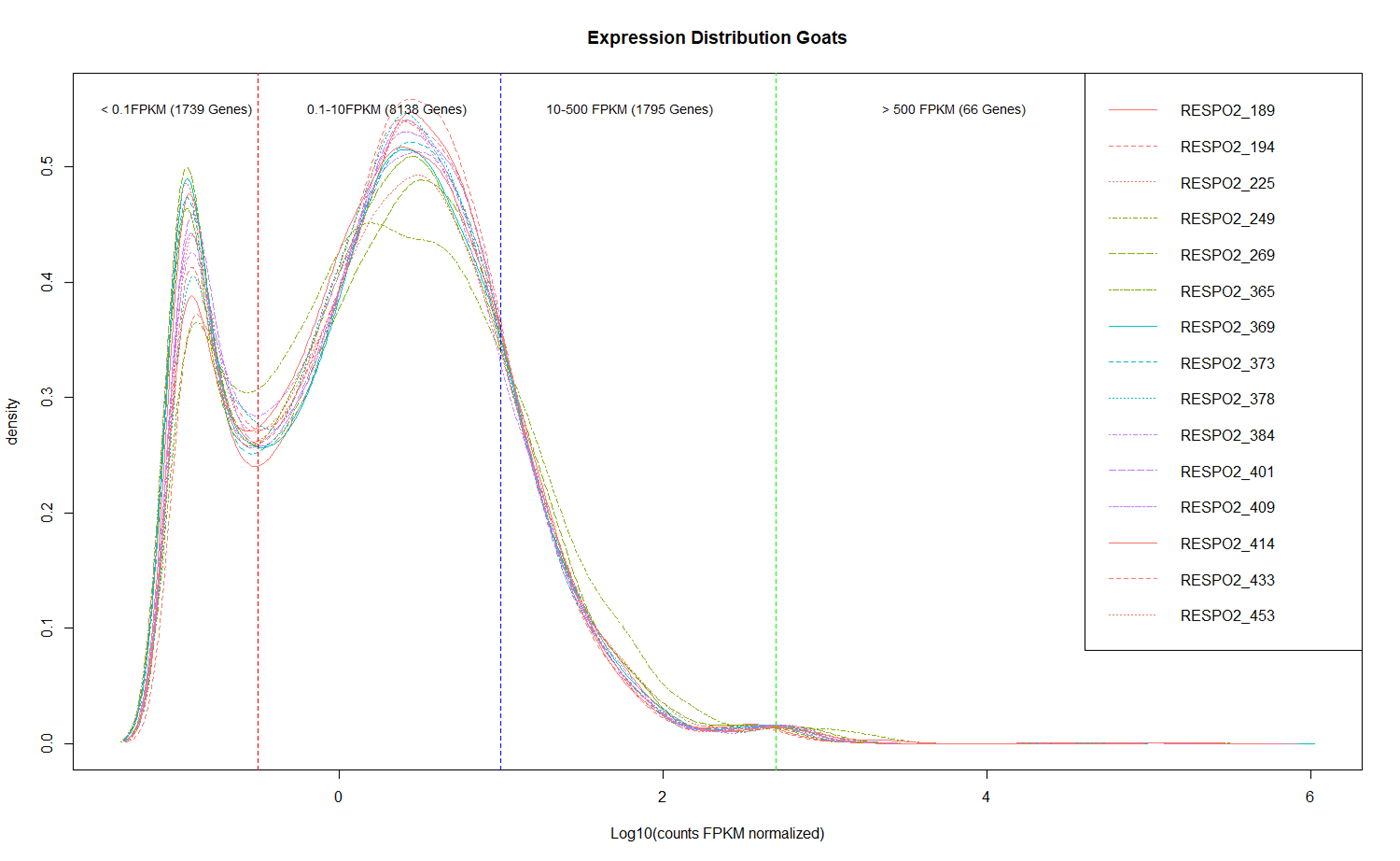

Supplement: Supplementary file 13 [file Image_1.png]
